# *Veillonella* and *Bacteroides* are associated with gestational diabetes mellitus exposure and gut microbiota immaturity

**DOI:** 10.1371/journal.pone.0302726

**Published:** 2024-05-14

**Authors:** Fernanda Valdez-Palomares, Jaqueline Reyes Aguilar, Eduardo Pérez-Campos, Laura Pérez-Campos Mayoral, Noemi Meraz-Cruz, Berenice Palacios-González

**Affiliations:** 1 Laboratorio de Envejecimiento Saludable, Instituto Nacional de Medicina Genómica, Centro de Investigación Sobre Envejecimiento (CIE-CINVESTAV Sur), Ciudad de México, México; 2 Hospital de la Niñez Oaxaqueña, Oaxaca, México; 3 Unidad de Bioquímica e Inmunología, Tecnológico Nacional de México-Instituto Tecnológico de Oaxaca, Oaxaca, México; 4 Centro de Investigación Facultad de Medicina UNAM-UABJO, Facultad de Medicina y Cirugía, Universidad Autónoma “Benito Juárez” de Oaxaca, Oaxaca, México; 5 Unidad de Vinculación Científica de la Facultad de Medicina UNAM en Instituto Nacional de Medicina Genómica, Ciudad de México, México; Max Delbruck Centrum fur Molekulare Medizin Berlin Buch, GERMANY

## Abstract

**Background:**

Dysbiosis during childhood impacts the configuration and maturation of the microbiota. The immaturity of the infant microbiota is linked with the development of inflammatory, allergic, and dysmetabolic diseases.

**Aims:**

To identify taxonomic changes associated with age and GDM and classify the maturity of the intestinal microbiota of children of mothers with GDM and children without GDM (n-GDM).

**Methods:**

Next-generation sequencing was used to analyze the V3–V4 region of 16S rRNA gene. QIIME2 and Picrust2 were used to determine the difference in the relative abundance of bacterial genera between the study groups and to predict the functional profile of the intestinal microbiota.

**Results:**

According to age, the older GDM groups showed a lower alpha diversity and different abundance of Enterobacteriaceae, *Veillonella*, *Clostridiales*, and *Bacteroides*. Regarding the functional profile, PWY-7377 and K05895 associated with Vitamin B12 metabolism were reduced in GDM groups. Compared to n-GDM group, GDM offspring had microbiota immaturity as age-discriminatory taxa in random forest failed to classify GDM offspring according to developmental age (OOB error 81%). Conclusion. Offspring from mothers with GDM have a distinctive taxonomic profile related to taxa associated with gut microbiota immaturity.

## Introduction

Gestational diabetes mellitus (GDM) according to the American Diabetes Association is defined as “diabetes diagnosed in the second or third trimester of pregnancy that was not clearly overt diabetes before gestation” [1]. In Mexico, the prevalence of GDM has been increasing; currently, its incidence is 17.7% [2]. GDM during pregnancy increases the susceptibility of offspring to develop insulin resistance, obesity, and hypertension [3,4].

Early alteration of the childhood microbiota is associated with allergies, inflammation, and childhood obesity [5–7]. According to the Developmental Origin of Health and Disease (DOHaD) theory the intrauterine exposure to excessive energy may result in permanent physiological and metabolic alterations, increasing disease risk in adulthood to developing obesity and type 2 diabetes [8–13].

The intestinal microbiota of the newborn is particularly interesting because, due to rapid temporal variation, the bacterial communities in the intestine are remarkably unstable. Therefore, early childhood is a crucial time window where the child’s gut microbiota can be modified [14,15] in contrast to an adult individual’s "mature" microbiota, which seems relatively stable over time.

The aim of this study was to identify taxonomic changes associated with age and GDM and to classify the gut-microbiota maturity of offspring from mothers with GDM and the offspring from mothers without GDM (n-GDM)

## Materials and methods

### Study population

The present is a cross-sectional study conducted in “C.S.T.III Dr. Gabriel Garzón Cossa" and “Hospital de la Niñez Oaxaqueña” located in Mexico. Offspring exposed and not exposed to GDM aged 0 to 30 months, either born vaginally or *via* C-section were included during routine hospital visit, only mothers that underwent a 75-g oral glucose tolerance test (OGTT) between 24- and 28-weeks’ gestation as part of screening protocol to determine GDM diagnosis were included. GDM was diagnosed according to the International Association of the Diabetes and Pregnancy Study Group criteria: Plasma glucose level ≥8.5 mmol/L following a 75 g OGTT [16]. A total of 40 infants were included: 26 infants not exposed to GDM (0–6 Months (n = 6), 7–12 Months (n = 10) and 13–30 Months (n = 10)) and 14 infants exposed to GDM (0–6 Months (n = 4), 7–12 Months (n = 5) and 13–30 Months (n = 5)). The 2009 WHO child growth standards (World Health Organization 2009) were used as a reference for children’s weight and length/height [17]. Maternal and infant characteristics and feeding practices (breastfeeding, formula, or mixed) were collected from medical records during interview and medical records (data were accessed for research purposes between the 9th of January 2017 and the 30th of November 2017). The following exclusion criteria were used: Infants with history of hospitalization and or use of antibiotics six months before the study, presence of chronic illness, gastrointestinal pathology, or diarrheal illness presented one month before the study. The study was performed according to the latest version of the Declaration of Helsinki and was approved by the Human Research Ethical Committee of Universidad Nacional Autónoma de México 079/2017/CEI-HGT. All parents or legal guardians and children provided written informed consent.

### Anthropometric measurements

The children were weighed, for which their clothing, diapers, and ornaments were removed. Length was measured using an infantometer, placing the child in a supine position with the head resting against the headboard; the measurer slid the stirrup along the base until it was flat against the soles of the feet, and the measurement was recorded from the digital counter (accuracy ±1.0 mm). Head circumference was measured using Seca 121 tape (Seca 212). The anthropometric measurements were obtained using standardized procedures applied by trained personnel.

### Fecal bacterial DNA isolation, libraries preparation and bioinformatic analysis

Fecal samples from infants aged 0–6 Months: (n = 6 not exposed to GDM; n = 4 exposed to GDM), aged 7–12 Months: (n = 10 not exposed to GDM; n = 5 exposed to GDM) and aged 13–30 Months: (n = 10 not exposed to GDM; n = 5 exposed to GDM) were collected from diapers or digital rectal examination, and placed in a sterile plastic container. The QIAamp PowerFecal Pro DNA kit (QIAGEN) was used to isolate bacterial DNA. DNA concentrations were determined using a NanoDrop V3.8.1 spectrophotometer. The 16s rRNA gene was amplified using primers 338F and 806R, targeting the V3-V4 hypervariable regions. The libraries were sequenced at the Sequencing Unit of the Instituto Nacional de Medicina Genómica using the Illumina Miseq platform (Illumina, San Diego, CA) [18]. Fastq reads were processed using the Quantitative Insights Into Microbial Ecology 2 (QIIME 2) [19]. The dada2 denoise-paired instruction was used for denoising quality, chimera checking, and clustering. For the 97% taxonomic assignment, the SILVA 16S reference database (version_138) and the classifiers naïve Bayes algorithm. A rooted phylogenetic tree was generated for further statistical analysis for α-diversity tests and Bray-Curtis for β-diversity tests. Microbial community metagenome prediction was performed with PICRUSt2 [20]. Microbiome Analyst was used to create the heat trees with mean abundance and the non-parametric Wilcoxon rank sum test corrected by Benjamin Hochberg (FD) (http://www.microbiomeanalyst.ca) [21]. Raw data are available in https://www.ncbi.nlm.nih.gov/bioproject/ with the accession number: PRJNA1085791.

### Classification of gut-microbiota maturity by developmental stages using Random Forests

The random forest classification model used the ’randomForest’ R package. A rarefied ASV table served as input data; the model was trained on 26 non-GDM infants and validated on 10 non-GDM infants, built using the following parameters: ntree = 10,000 and mtry of p/3 ASVs randomly sampled at each split, in which p represents the number of ASVs. The model was further refined by applying tenfold cross-validation to estimate the minimal number of top-ranking age-discriminatory taxa required for prediction, including 15 ASVs to train the final model based on a mean decrease in Gini. The Random Forests algorithm was applied to classify healthy infants by chronologic age ranging from 0–6 months, >6–12 months, and >12–30 months, thereby identifying taxa that discriminate by chronologic age in healthy children and subsequently applied on 16 GDM offspring infants.

### Statistical analysis

Continuous quantitative variables (anthropometric data) will be presented as the mean ± standard deviation (SD) for variables with parametric distribution and median and 25th and 75th percentiles for variables with non-parametric distribution. To evaluate the differences between the study groups, the one-way ANOVA or Kruskall Wallis test will be used according to the homoscedasticity criterion and the normality of the data. Statistical analyses will be performed in the GraphPad 9 statistical program and R (https://www.r-project.org/) [22,23].

## Results

### Population demographics and clinical characteristics

A total of 40 children were included: 26 children not exposed to GDM (n-GDM) and 14 children exposed to GDM (GDM) (**Table 1)**. Regarding clinical parameters, there were no significant differences between the groups.

**Table 1:** Clinical characteristics.

Characteristics	n-GDM (n = 26)	GDM (n = 14)	p value
Mother age (y)	31.5 ± 6.1	31.8 ± 6.8	0.440
Number of pregnancies (n)	2 (1–3)	2 (1–3)	0.664
C-section delivery, n (%)	18 (69)	10 (71)	-
Gestational age (weeks)	38 (37–40)	38 (33–40)	0.125
Male sex, n (%)	19 (68)	16 (57)	-
Birth weight (g)	2752 ± 599	2875 ± 923	0.457
Birth height (cm)	47.5 ± 6.1	47.8 ± 4.5	0.526
Breastfeeding (%)	92	86	-
Breastfeeding duration (months)	7.7 ± 5.4	6.5 ± 4.9	0.457

Continuous variables compared by Wilcoxon rank-sum test and categorical variables by Fisher’s exact test.

p < 0.05.

### Composition of the bacterial community of children exposed to GDM

**Fig 1:**
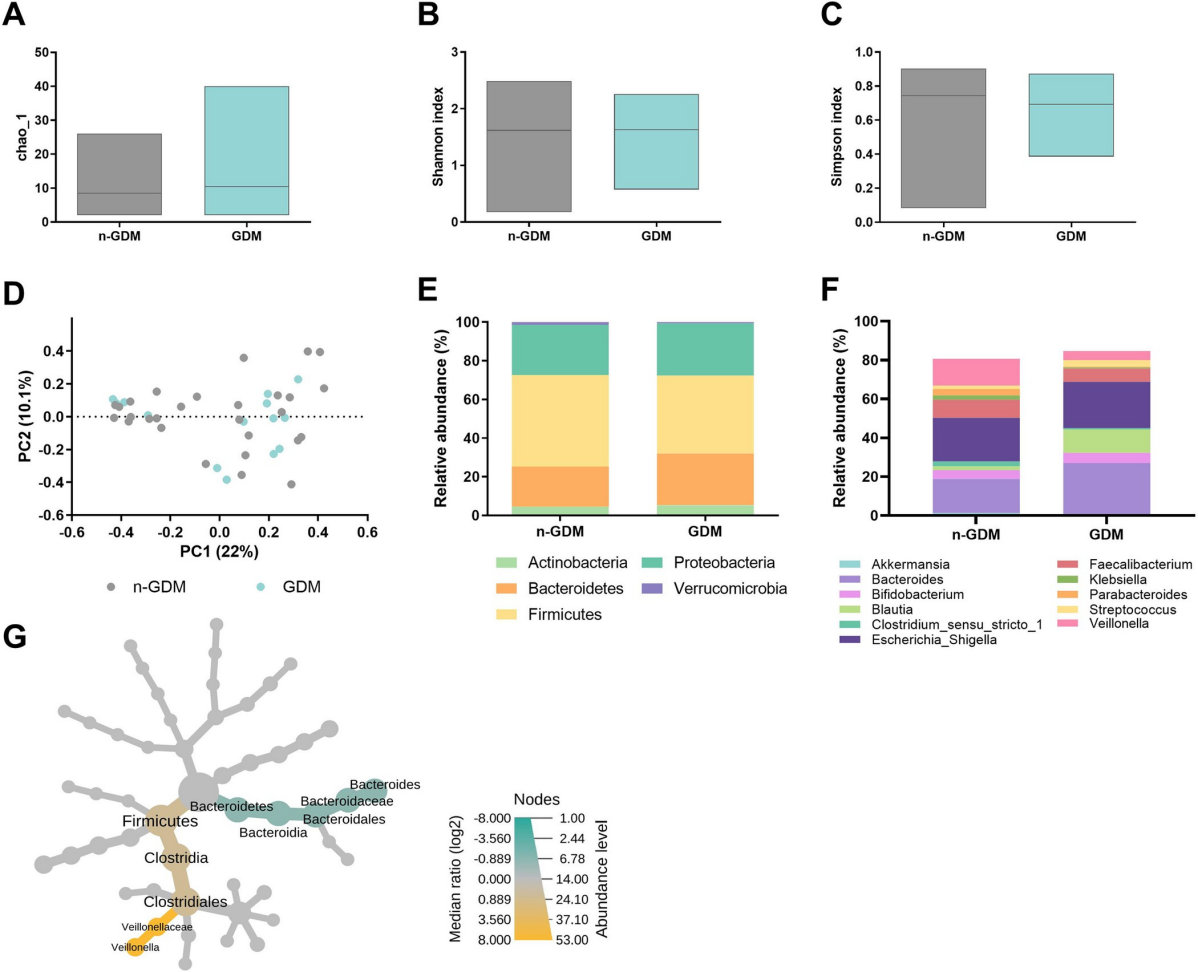
Microbiota analysis in children not exposed and exposed to GDM. (a) Chao index in n-GDM and GDM group, no significant differences were observed between the groups; (b) Shannon index in n-GDM and GDM group, no statistically significant differences were found between the groups; (c) Simpson index in n-GDM and GDM group, no differences were found among the group; (d) Bray-Curtis index in n-GDM and GDM groups, no significant differences were shown amongst the groups, n-GDM group (light blue circles) and GDM group (gray circles); (e) Phylum-level composition (% relative abundances) among the study groups. Firmicutes, coloured yellow, followed by Bacteroidetes, coloured orange; (f) Genus-level composition (% relative abundances) among the study groups, the most abundant taxa were *Bacteroides*, *Akkermansia*, *Bifidobacterium*, *Blautia*, *Clostridium*, *Escherichia_Shigella*, *Faecalibacterium*, *Klebsiella*, *Parabacteroides*, *Streptococcus* and *Veillonella*; (g) Heat tree for pair-wise comparison. Those taxa that showed statistically significant differences were *Veillonella* of yellow colour, enrichment in the n-GDM group; and green-coloured *Bacteroides* enrichment in the GDM group.

From the Illumina 330 bp paired-end sequencing of the amplicon targeting the V3–V4 region of 16S rRNA gene, two thousand five hundred seventy-nine high-quality sequences among the 30 fecal samples from the participants with an average of 30,027 sequences per sample were generated. Chao1, Shannon, and Simpson index were used to describe alpha diversity (**Fig 1A–1C**). There were no significant differences in alpha diversity between n-GDM and GDM groups. To compare microbial communities’ composition, we calculated the beta diversity by Bray-Curtis index (**Fig 1D**). No significant difference was found between the groups.

The predominant phyla were Firmicutes, with a mean abundance of 44%, followed by 24% of Bacteroidetes, Proteobacteria, which represented 26%, while Verrucomicrobia and Actinobacteria represented 1% and 5%, respectively (**Fig 1E**). Comparisons of the relative abundance of the taxa were made to determine differences at the taxonomic level between each age group (Fig 1F). The fecal microbiota of the GDM group had significantly lower (p = 0.007) proportions of Firmicutes, specifically in *Veillonella* genus, and significantly higher (p = 0.014) proportions of Bacteroidetes, specifically in *Bacteroides*, compared to n-GDM group (**Fig 1G**). Fecal microbiota according to type of delivery was also compared. In vaginally born infants, the predominant phyla were *Bacteroidetes*, while, *Proteobacteria* and *Actinobacteria* were main constituents in C-section infants (**S1A Fig**). No significant differences in alpha or beta diversity were detected among groups (**S1B and S1C Fig**), Enrichment in members of the *Proteobacteria* phyla: *Gammaproteobacteria*, *Enterobacteriales*, *Enterobacteriaceae* and *Escherichia_Shigella* genus was detected in C-section infants, while enrichment in the *Bacteroidetes* phylum members, *Bacteroides*, *Bacteroidales*, *Bacteroidaceae* and *Bacteroides* was detected in vaginally born infants (**S1D Fig**).

### Influences of age-related GDM on the microbiota composition

**Fig 2:**
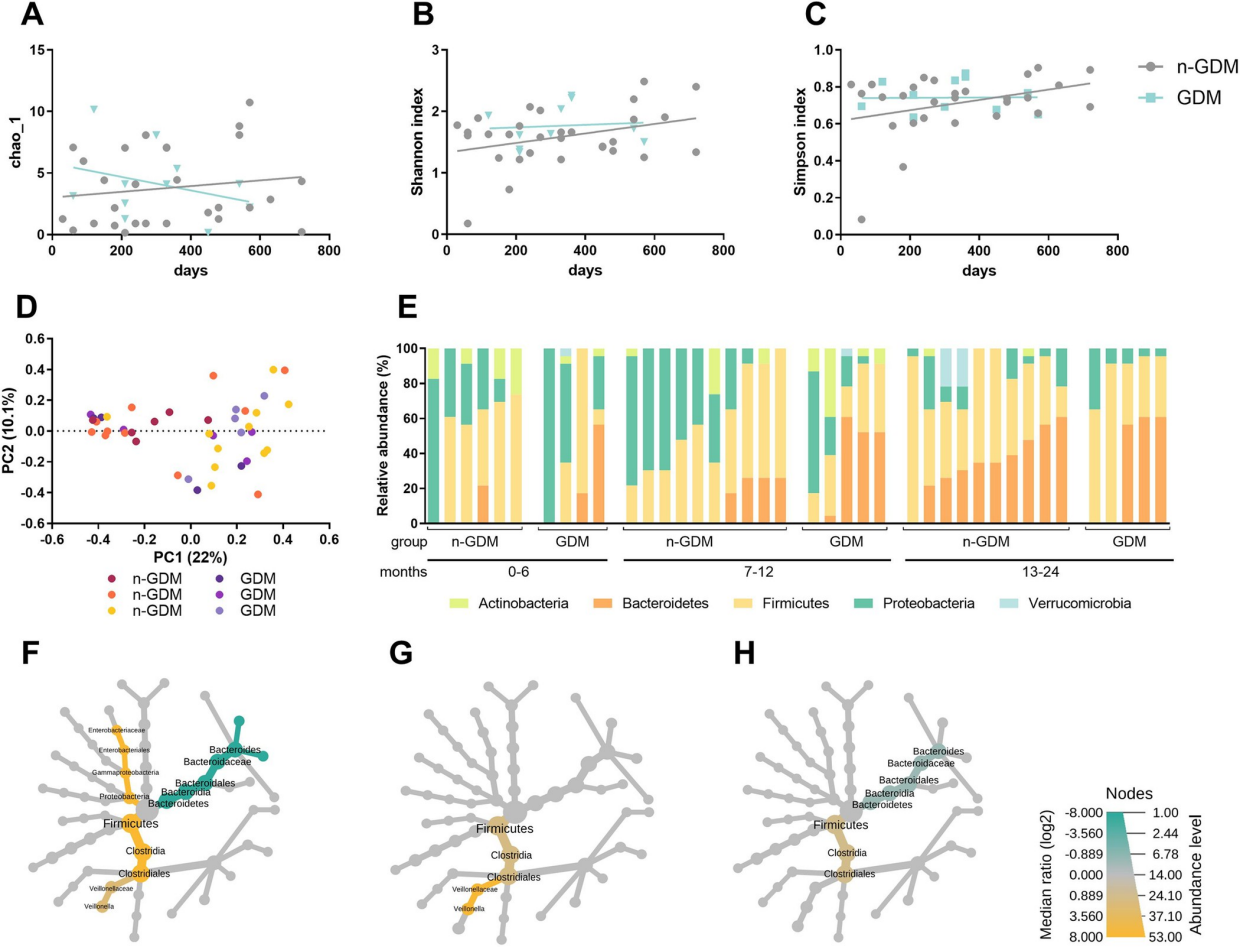
Impacts of age-related GDM on the microbiota composition. (a) Chao index, (b) Shannon index, (c) Simpson index across the age, n-GDM group (light blue circles) and GDM group (gray circles), the GDM group presented lower alpha diversity as age advance; (d) Bray-Curtis index across the age in n-GDM and GDM groups, no significant differences were found between the groups (F-value: 1.2489; R2: 0.14782; p-value: 0.088), newborns to 6 months (n-GDM group, red circles; GDM group, dark purple), 7 to 12 months (n-GDM group, orange circles; GDM group, light purple) and 13 months to 24 months (n-GDM group, yellow circles; GDM group, gray circles); (e) Phylum-level composition (% relative abundances) among the study groups; Heat tree for pair-wise comparison, divided by age, (f) newborns to 6 months, (g) 7 to 12 months and (h) 13 months to 24 months. Those taxa that showed statistically significant differences are shown in colour. Taxa coloured yellow are enriched in the n-GDM group and those coloured green are enriched in the GDM group. The colour of each taxon indicates the log-2 ratio of the proportions observed in each condition.

The effect of age on the bacterial community composition was assessed. Regarding alpha diversity, the GDM group presented lower alpha diversity as age advanced (**Fig 2A–2C**). Interestingly, the GDM group remains at all alpha diversity metrics below the n-GDM groups. The beta diversity analysis **(Fig 2D**), calculated on the Bray-Curtis dissimilarity, revealed no statistically significant difference (F-value: 1.2489; R2: 0.14782; p-value: 0.088). Subsequently, the groups were divided according to age ranges: newborns to 6 months (Group 1), 7 to 12 months (Group 2), and 13 months to 24 months (Group 3). At the phyla level, no significant differences were found (**Fig 2E**). On the other hand, the GDM-1 group presented a lower abundance of Enterobacteriaceae (family) (**Fig 2F**). The GDM-1 and GDM2 groups showed a lower abundance of *Veillonella* (a genus belonging to the Clostridiales order) (**Fig 2F and 2G**), while the GDM3 group only showed a lower abundance in Clostridiales without reaching a difference in any specific genus (**Fig 2H**). The GDM-1 and GDM-3 groups also presented a higher abundance of *Bacteroides* (genus) (**Fig 2F and 2G)**.

### Age-related effects on microbial functional pathways

**Fig 3:**
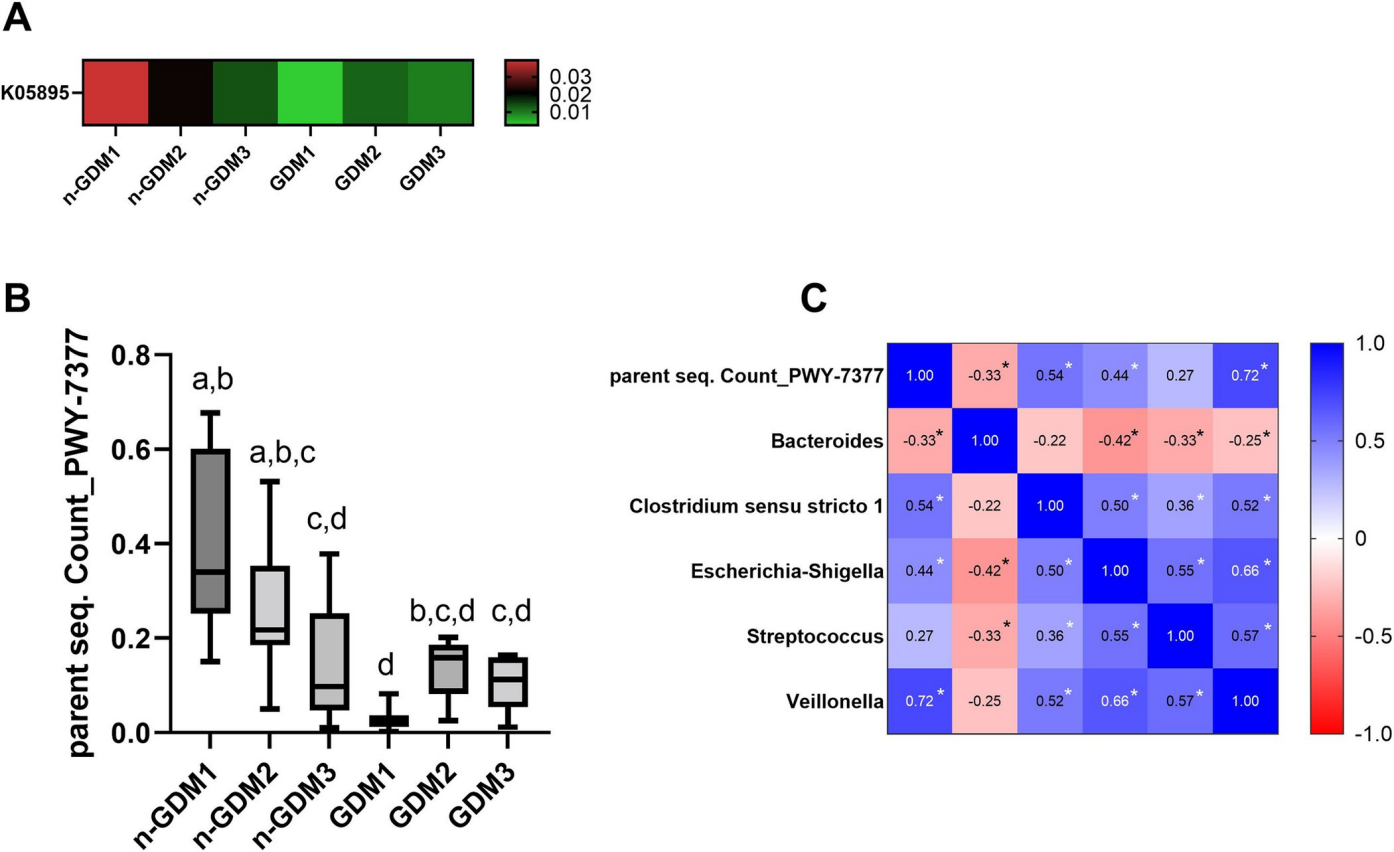
Metagenomic predictions of the gut microbiota of the n-GDM and GDM groups. (a) KEGG Orthology (KO; K05895). Red indicates enrichment and green shows depletion; (b) Microbial metabolic pathway PWY-7377 Cob(II)yrinate a,c-diamide bio-synthesis I (early cobalt insertion) in n-GDM and GDM groups. p <0.05 are considered statistically significant differences. Statistically significant differences among the groups are shown with letters, where a>b>c>d; (c) Heatmap correlogram containing Pearson’s rho negative and positive correlation coefficients. Blue and red color denotes positive and negative correlation coefficients correlation, respectively. *p < 0.05. Fecal samples from infants aged 0–6 Months: (n-GDM1: n = 6; GDM1: n = 4), aged 7–12 Months: (n-GDM2: n = 10; GDM2: n = 5) and aged 13–30 Months: (n-GDM3: n = 10; GDM3: n = 5).

PICRUSt2 was employed to predict functional differences in fecal microbiota. The n-GDM microbiome was enriched for the KEGG pathway oxidoreductases (K05895 (cobK-cbiJ); pre-corrin-6A/cobalt-pre corrin-6A reductase; Vitamin B12 metabolism) and PWY-7377 (adenosylcobalamin biosynthesis I (anaerobic); cob(II) urinate a c-diamide biosynthesis I (early cobalt insertion)) (**Fig 3A and 3B**). These pathways were inversely related to age in n-GDM group. Vitamin 12 biosynthesis is mediated exclusively by the bacterial fermentation process, so a correlation matrix between the predicted abundance pathway and differentially expressed taxa was performed. PWY-7377 positively correlated with *Veillonella*, *Clostridium sensu stricto*; *Escherichia-Shigella* and *Streptococcus*, meanwhile negatively correlated with *Bacteroides* (**Fig 3C**).

### GDM infants have immature gut microbiota in the early stages of development

**Fig 4:**
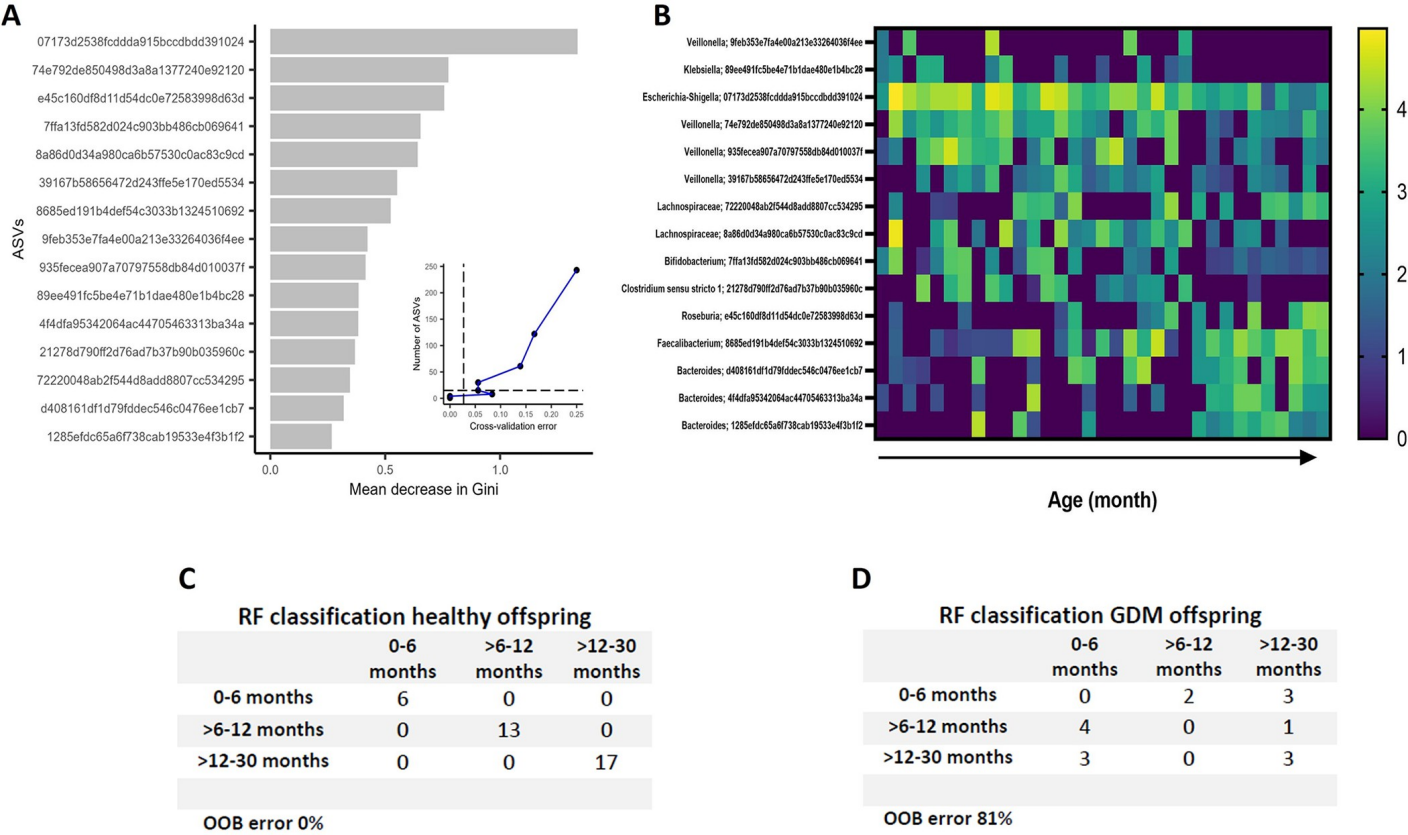
Maturation of the microbiota. A top 15 age-discriminatory taxa random forest classification model was constructed with healthy offspring samples. (a) The number of features was selected by ten-fold cross-validation (b). The 15 features in the model and their abundance are shown the model was applied to healthy offspring (c) and to GDM offspring (d).

To define the maturity of the microbiota, the Random Forests (RF) model resulting from healthy children was used, thus determining those taxa that help to distinguish the different stages of postnatal life. Rated lists of all bacterial taxa, in order of ’age-discriminative importance,’ were selected by considering those whose relative abundance values, when permuted, have the most considerable mean decrease in Gini. An increase in validation error was observed when including taxa beyond the top 15 (**Fig 4A**). Log-transformed counts of the top 15 relatives of age-discriminating taxa are shown in **Fig 4B**. RF model of 15 top discriminatory taxa accurately classified healthy offspring according to developmental age (OOB error 0%) (**Fig 4C**). The RF model was later applied with no further parameter optimization to classify GDM offspring according to periods of postnatal life. The results revealed that compared to healthy, GDM offspring had microbiota immaturity as age-discriminatory taxa in RF failed to classify developmental age (OOB error 81%) of GDM offspring (**Fig 4D**).

## Discussion

GDM is a public health problem that increases the risk of maternal and perinatal morbidity and mortality and contributes to the development of long-term health complications in both mothers and offspring. GDM has been associated with changes in the intestinal microbiota both in the neonate and during pregnancy [24–27]. Interestingly, intestinal dysbiosis during the first stages of life is associated with long-term health status, which denotes the importance of the intestinal microbiota in the first years of life on the development of metabolic and immunological diseases [28,29]. In the present study, no changes in microbiota composition were observed when comparing children exposed to GDM with children not exposed to GDM. However, when the groups were separated by age, the offspring of mothers with GDM maintained lower alpha diversity. As mentioned above, the gut microbiota in early childhood (≤2 years) changes over time [30], both at the taxonomic level and in alpha and beta diversity metrics [31]. Offspring that were not exposed to DMG showed continuous development and maturation of the intestinal microbiota during the first years of life, unlike offspring from mothers exposed to DMG that presented lower alpha diversity, which has been associated with overall delay in microbial maturation and subsequent adverse health outcomes, including atopy and allergic diseases [32,33]. This result is consistent with other studies indicating that GDM is associated with lower diversity [34,35]. Unlike what Crusell et al. observed, alpha diversity remains decreased in the offspring of children exposed to GDM and does not show the recovery observed at nine months [34]. Furthermore, obese children have less diversity and richness [36], suggesting that in children with GDM, the immaturity of the gut microbiota could be a determining factor for the development of obesity in the future [37].

Several studies suggest early colonization is essential for establishing and maturing the gut microbiota. During the early colonization of the newborn intestine, facultative anaerobic Proteobacteria gradually consume oxygen in the gastrointestinal tract, and, consequently, obligate anaerobes colonize the new environment [38]. Premature infants exhibit immaturity in the gut microbiota, characterized by the predominance of pathogenic bacteria within the Enterobacteriaceae family, persistently low diversity, and a scarcity of strictly anaerobic taxa, including *Veillonella* relative to appropriately growing [39]. Interestingly, in this study, the offspring of mothers with GDM showed a lower abundance, specifically of the taxon *Veillonella*, which, as mentioned above, is considered involved in developing the gut microbiota as seed species along with Streptococcus [40]. The above suggests that the presence of GDM affects the maturity of the intestinal microbiota.

In addition, the groups with GDM had a greater abundance of Bacteroides; Vatanen et al. demonstrated *in vitro* studies that *Bacteroides* species from newborns with increased susceptibility to type 1 diabetes and allergies produce a subtype of lipopolysaccharide (LPS) that inhibits the immunostimulatory activity of *Escherichia coli* LPS [41]. The authors also point out that in the first year of life, the metabolism of human milk oligosaccharides (HMO) is involved in the maintenance and/or establishment of an intestinal microbiota dominated by Bifidobacterium versus Bacteroides, presumably because both genera contend for the HMO as an energy source, the above is of clinical relevance to promote breastfeeding in children from pregnancies with GDM. Differential enrichment in *Bacteroidetes*- and *Proteobacteria*-related taxa according to type of delivery was detected in infants as previously reported by other authors [42]. However, the present RF classification model trained using data from infants with different type of delivery, was conducted to define chronological age and microbiota maturity in the offspring form healthy mothers regardless of the type of delivery. According to this, ASVs assigned to the discriminatory genera *Escherichia_Shigella* and *Bacteroides* in C-section and vaginally born infants were among top 15 age discriminatory taxa in n-GDM offspring group, therefore, discriminatory taxa for type of delivery can also define microbiota maturity.

On the other hand, in this study, the PWY-7377 pathway implicated in the biosynthesis of vitamin B12 (cobalamin) was inversely related to age in the n-GDM group and significantly correlated with the abundance of *Veillonella*. The synthesis of vitamin B12 is mainly carried out by three orders: Propionibacterales, Corynebacterales, Coriobacterales belonging to Actinobacteria, and the orders Clostridiales, Selenomonadales and Veillonellales (*Veillonella atypica* and *Veillonella parvula*) of Firmicutes [43]. Although cobalamin in mammals is synthesized exclusively by the gut microbiota, microbial vitamin B12 production plays a limited function due to its limited availability in the environment. Two issues prevent humans from obtaining significant levels of cobalamin from this source. First, of the total corrinoids in feces, less than 2% of cobalamin is produced by intestinal bacteria; Secondly, due to the absence of transporters of this vitamin in the colon [44,45]. Taking this into account, a possible explanation for our findings could come from vitamin B12-dependent reactions in bacteria. Vitamin B12 contributes to the microbiota as a modulator of intestinal microbial ecology. Since 80% of bacteria encode cobalamin-dependent enzymes [45]. Considering this information and our results, one hypothesis is that those prototrophic taxa, such as *Veillonella*, would help supply cobalamin to auxotrophic taxa during the first months of life. Kundra et al. demonstrated that even in the absence of B12, the presence of prototrophic bacteria could supply sufficient cobalamin for the remaining 80% of auxotrophic taxa [46]. Thus, the presence of *Veillonella* could help modulate the configuration of the microbiota in the early stages of life.

We found that GDM-exposed offspring had disrupted microbiota maturation characterized by persistently low diversity and reduced *Veillonella* compared to non-GDM-exposed infants. Finally, a random forest classification model was used to model the maturation of the microbiota of healthy infants, based on the approach of Subramanian et al. [47]. The model taxa included *Bifidobacterium*, *Escherichia-Shigella*, *Bacteroides*, *Veillonella*, *Clostridium sensu stricto* (group I), and *Faecalibacterium*. Interestingly, *Bacteroides*, *Bifidobacterium*, and *Clostridium sensu stricto I are*first colonizers of the infant intestine [48]. While *Faecalibacterium prausnitzii* is present at low abundance and, in some cases, absent during early childhood and with advancing age, its abundance increases until reaching the levels observed in adulthood [49], suggesting that GDM negatively affects the early establishment in the gut of early colonizers concerning health and delayed establishment of microbiota members that are expected to increase in abundance after the first year of life. However, limitations of the study, are the relatively small number of infants exposed and not exposed to GDM and the predictive limitations of our cross-sectional study, additional prospective studies are needed to replicate our findings and determine whether these signatures of microbiota maturation could apply to GDM-exposed offspring. However, limitations of the study, are the relatively small number of infants exposed and not exposed to GDM and the predictive limitations of a cross-sectional study, further prospective studies are needed to replicate our findings and determine whether these signatures of microbiota maturation could be applicable to GDM-exposed offspring.

## Conclusion

In conclusion, children of mothers with GDM have a distinctive taxonomic profile related to the immaturity of the intestinal microbiota. Our results show the importance of the first months of life for the maturation and future configuration of the intestinal microbiota.

## Supporting information

S1 Fig Microbiota analysis in children not exposed and exposed to GDM stratified by pregnancy outcome. (a) Phylum-level composition (% relative abundances) among the study groups. (b) Observed features, Chao and Shannon index in C-section and vaginally born infants, no significant differences were observed between the groups; (c) Bray-Curtis index in C-section and vaginally born infants, no significant differences were shown amongst the groups. (d) Heat tree for pair-wise comparison. Those taxa that showed statistically significant differences were members of *Proteobacteria* in C-section group (red) and members of *Bacteroidetes* in vaginally born infants group (blue). The colour of each taxon indicates the log-2 ratio of the proportions observed in each condition.
(TIF)

## References

[1] **<**References>. 2. Classification and Diagnosis of Diabetes: Standards of Medical Care in Diabetes-2022. Diabetes Care, 2022. 45(Suppl 1): p. S17–s38. doi: 10.2337/dc22-S002 34964875

[2] Reyes-Munoz E., et al., Effect of the diagnostic criteria of the International Association of Diabetes and Pregnancy Study Groups on the prevalence of gestational diabetes mellitus in urban Mexican women: a cross-sectional study. Endocr Pract, 2012. 18(2): p. 146–51. doi: 10.4158/EP11167.OR 21856596

[3] Lowe W.L., Jr., et al., Maternal BMI and Glycemia Impact the Fetal Metabolome. Diabetes Care, 2017. 40(7): p. 902–910. doi: 10.2337/dc16-2452 28637888 PMC5481987

[4] Tam W.H., et al., In Utero Exposure to Maternal Hyperglycemia Increases Childhood Cardiometabolic Risk in Offspring. Diabetes Care, 2017. 40(5): p. 679–686. doi: 10.2337/dc16-2397 28279981 PMC5399651

[5] Munyaka P.M., Khafipour E., and Ghia J.E., External influence of early childhood establishment of gut microbiota and subsequent health implications. Front Pediatr, 2014. 2: p. 109. doi: 10.3389/fped.2014.00109 25346925 PMC4190989

[6] Wallace J.G., Gohir W., and Sloboda D.M., The impact of early life gut colonization on metabolic and obesogenic outcomes: what have animal models shown us? J Dev Orig Health Dis, 2016. 7(1): p. 15–24. doi: 10.1017/S2040174415001518 26399435

[7] Rautava S., Microbial Composition of the Initial Colonization of Newborns. Nestle Nutr Inst Workshop Ser, 2017. 88: p. 11–21. doi: 10.1159/000455209 28346920

[8] Hoffman D.J., Reynolds R.M., and Hardy D.B., Developmental origins of health and disease: current knowledge and potential mechanisms. Nutr Rev, 2017. 75(12): p. 951–970. doi: 10.1093/nutrit/nux053 29186623

[9] Lin X., et al., Developmental pathways to adiposity begin before birth and are influenced by genotype, prenatal environment and epigenome. BMC Med, 2017. 15(1): p. 50. doi: 10.1186/s12916-017-0800-1 28264723 PMC5340003

[10] Macaulay E.C., et al., The importance of early life in childhood obesity and related diseases: a report from the 2014 Gravida Strategic Summit. J Dev Orig Health Dis, 2014. 5(6): p. 398–407. doi: 10.1017/S2040174414000488 25308169 PMC4255318

[11] El Hajj N., et al., Epigenetics and life-long consequences of an adverse nutritional and diabetic intrauterine environment. Reproduction, 2014. 148(6): p. R111–20. doi: 10.1530/REP-14-0334 25187623 PMC4241689

[12] Fleisch A.F., et al., Prenatal and early life exposure to traffic pollution and cardiometabolic health in childhood. Pediatr Obes, 2017. 12(1): p. 48–57. doi: 10.1111/ijpo.12106 26843357 PMC4974151

[13] Perng W., et al., Birth Size, Early Life Weight Gain, and Midchildhood Cardiometabolic Health. J Pediatr, 2016. 173: p. 122–130 e1. 26995700 10.1016/j.jpeds.2016.02.053 PMC4884526

[14] Renz H., et al., The neonatal window of opportunity-early priming for life. J Allergy Clin Immunol, 2018. 141(4): p. 1212–1214. doi: 10.1016/j.jaci.2017.11.019 29247715 PMC6214658

[15] Koletzko B., et al., Research and the promotion of child health: a position paper of the European Society for Pediatric Gastroenterology, Hepatology, and Nutrition. J Pediatr Gastroenterol Nutr, 2014. 59(2): p. 274–8. doi: 10.1097/MPG.0000000000000411 24796801

[16] Metzger B.E., et al., International association of diabetes and pregnancy study groups recommendations on the diagnosis and classification of hyperglycemia in pregnancy. Diabetes Care, 2010. 33(3): p. 676–82. doi: 10.2337/dc09-1848 20190296 PMC2827530

[17] Organization W.H., WHO child growth standards and the identification of severe acute malnutrition in infants and children: A Joint Statement by the World Health Organization and the United Nations Children’s Fund. 2009, World Health Organization: Geneva, Switzerland. 24809116

[18] Caporaso J.G., et al., Ultra-high-throughput microbial community analysis on the Illumina HiSeq and MiSeq platforms. ISME J, 2012. 6(8): p. 1621–4. doi: 10.1038/ismej.2012.8 22402401 PMC3400413

[19] Bolyen E., et al., Reproducible, interactive, scalable and extensible microbiome data science using QIIME 2. Nat Biotechnol, 2019. 37(8): p. 852–857. doi: 10.1038/s41587-019-0209-9 31341288 PMC7015180

[20] Douglas G.M., et al., PICRUSt2 for prediction of metagenome functions. Nat Biotechnol, 2020. 38(6): p. 685–688. doi: 10.1038/s41587-020-0548-6 32483366 PMC7365738

[21] Lu Y., et al., MicrobiomeAnalyst 2.0: comprehensive statistical, functional and integrative analysis of microbiome data. Nucleic Acids Res, 2023. doi: 10.1093/nar/gkad407 37166960 PMC10320150

[22] Team R.C. R: A language and Environment for Statistical Computing, v. 3.0.0.. 2013; Available from: https://www.R-project.org/.

[23] Team R.C. R Package Stats: A Language and Environment for Statistical Computing. 2015; Available from: https://www.R-project.org/.

[24] Wang J., et al., Dysbiosis of maternal and neonatal microbiota associated with gestational diabetes mellitus. Gut, 2018. 67(9): p. 1614–1625. doi: 10.1136/gutjnl-2018-315988 29760169 PMC6109274

[25] Ponzo V., et al., The microbiota composition of the offspring of patients with gestational diabetes mellitus (GDM). PLOS ONE, 2019. 14(12): p. e0226545. doi: 10.1371/journal.pone.0226545 31841548 PMC6913919

[26] Hasan S., et al., Gut microbiome in gestational diabetes: a cross-sectional study of mothers and offspring 5 years postpartum. Acta Obstet Gynecol Scand, 2018. 97(1): p. 38–46. 29077989 10.1111/aogs.13252

[27] Wang J., et al., Dysbiosis of maternal and neonatal microbiota associated with gestational diabetes mellitus. Gut, 2018. doi: 10.1136/gutjnl-2018-315988 29760169 PMC6109274

[28] Gabriel C.L. and Ferguson J.F., Gut Microbiota and Microbial Metabolism in Early Risk of Cardiometabolic Disease. Circ Res, 2023. 132(12): p. 1674–1691. doi: 10.1161/CIRCRESAHA.123.322055 37289901 PMC10254080

[29] Sarkar A., et al., The Association between Early-Life Gut Microbiota and Long-Term Health and Diseases. J Clin Med, 2021. 10(3). doi: 10.3390/jcm10030459 33504109 PMC7865818

[30] Butel M.J., Waligora-Dupriet A.J., and Wydau-Dematteis S., The developing gut microbiota and its consequences for health. J Dev Orig Health Dis, 2018: p. 1–8. doi: 10.1017/S2040174418000119 29562949

[31] Robertson R.C., et al., The Human Microbiome and Child Growth–First 1000 Days and Beyond. Trends in Microbiology, 2019. 27(2): p. 131–147. doi: 10.1016/j.tim.2018.09.008 30529020

[32] Fouhy F., et al., Perinatal factors affect the gut microbiota up to four years after birth. Nature Communications, 2019. 10(1). doi: 10.1038/s41467-019-09252-4 30944304 PMC6447568

[33] Stokholm J., et al., Maturation of the gut microbiome and risk of asthma in childhood. Nature Communications, 2018. 9(1). 10.1038/s41467-017-02573-2 PMC5762761 29321519

[34] Crusell M.K.W., et al., Comparative Studies of the Gut Microbiota in the Offspring of Mothers With and Without Gestational Diabetes. Frontiers in Cellular and Infection Microbiology, 2020. 10. doi: 10.3389/fcimb.2020.536282 33194786 PMC7645212

[35] Su M., et al., Diversified gut microbiota in newborns of mothers with gestational diabetes mellitus. PLOS ONE, 2018. 13(10): p. e0205695. doi: 10.1371/journal.pone.0205695 30332459 PMC6192631

[36] Yu Z., et al., Greater alteration of gut microbiota occurs in childhood obesity than in adulthood obesity. Front Pediatr, 2023. 11: p. 1087401. doi: 10.3389/fped.2023.1087401 36776907 PMC9909466

[37] Nash M.J., Frank D.N., and Friedman J.E., Early Microbes Modify Immune System Development and Metabolic Homeostasis-The "Restaurant" Hypothesis Revisited. Front Endocrinol (Lausanne), 2017. 8: p. 349. doi: 10.3389/fendo.2017.00349 29326657 PMC5733336

[38] Huang Y.E., et al., Disrupted establishment of anaerobe and facultative anaerobe balance in preterm infants with extrauterine growth restriction. Front Pediatr, 2022. 10: p. 935458. doi: 10.3389/fped.2022.935458 36147811 PMC9486202

[39] Younge N.E., et al., Disrupted Maturation of the Microbiota and Metabolome among Extremely Preterm Infants with Postnatal Growth Failure. Scientific Reports, 2019. 9(1). doi: 10.1038/s41598-019-44547-y 31160673 PMC6546715

[40] Reddel S., et al., A Parallel Tracking of Salivary and Gut Microbiota Profiles Can Reveal Maturation and Interplay of Early Life Microbial Communities in Healthy Infants. Microorganisms, 2022. 10(2): p. 468. doi: 10.3390/microorganisms10020468 35208921 PMC8880349

[41] Vatanen T., et al., Variation in Microbiome LPS Immunogenicity Contributes to Autoimmunity in Humans. Cell, 2016. 165(4): p. 842–53. doi: 10.1016/j.cell.2016.04.007 27133167 PMC4950857

[42] Mitchell CM., et al., Delivery Mode Affects Stability of Early Infant Gut Microbiota. Cell Rep Med. 2020; 1(9): p. 100156. doi: 10.1016/j.xcrm.2020.100156 33377127 PMC7762768

[43] Balabanova L., et al., Microbial and Genetic Resources for Cobalamin (Vitamin B12) Biosynthesis: From Ecosystems to Industrial Biotechnology. International Journal of Molecular Sciences, 2021. 22(9): p. 4522. doi: 10.3390/ijms22094522 33926061 PMC8123684

[44] Patrick Michiko, and Andrew, Vitamin B 12 as a Modulator of Gut Microbial Ecology. Cell Metabolism, 2014. 20(5): p. 769–778. 25440056 10.1016/j.cmet.2014.10.002 PMC4260394

[45] Rowley C.A. and Kendall M.M., To B12 or not to B12: Five questions on the role of cobalamin in host-microbial interactions. PLOS Pathogens, 2019. 15(1): p. e1007479. doi: 10.1371/journal.ppat.1007479 30605490 PMC6317780

[46] Kundra P., et al., Healthy adult gut microbiota sustains its own vitamin B12 requirement in an in vitro batch fermentation model. Front Nutr, 2022. 9: p. 1070155. doi: 10.3389/fnut.2022.1070155 36532531 PMC9751363

[47] Subramanian S., et al., Persistent gut microbiota immaturity in malnourished Bangladeshi children. Nature, 2014. 510(7505): p. 417–21. doi: 10.1038/nature13421 24896187 PMC4189846

[48] Milani C., et al., The First Microbial Colonizers of the Human Gut: Composition, Activities, and Health Implications of the Infant Gut Microbiota. Microbiol Mol Biol Rev, 2017. 81(4). doi: 10.1128/MMBR.00036-17 29118049 PMC5706746

[49] Yassour M., et al., Natural history of the infant gut microbiome and impact of antibiotic treatment on bacterial strain diversity and stability. Sci Transl Med, 2016. 8(343): p. 343ra81. doi: 10.1126/scitranslmed.aad0917 27306663 PMC5032909

