# Supplementary figures and images for: Veillonella and Bacteroides are associated with gestational diabetes mellitus exposure and gut microbiota immaturity
Source: PLoS One. 2024 May 14;19(5):e0302726. doi: 10.1371/journal.pone.0302726 (PMC11093295; doi:10.1371/journal.pone.0302726)

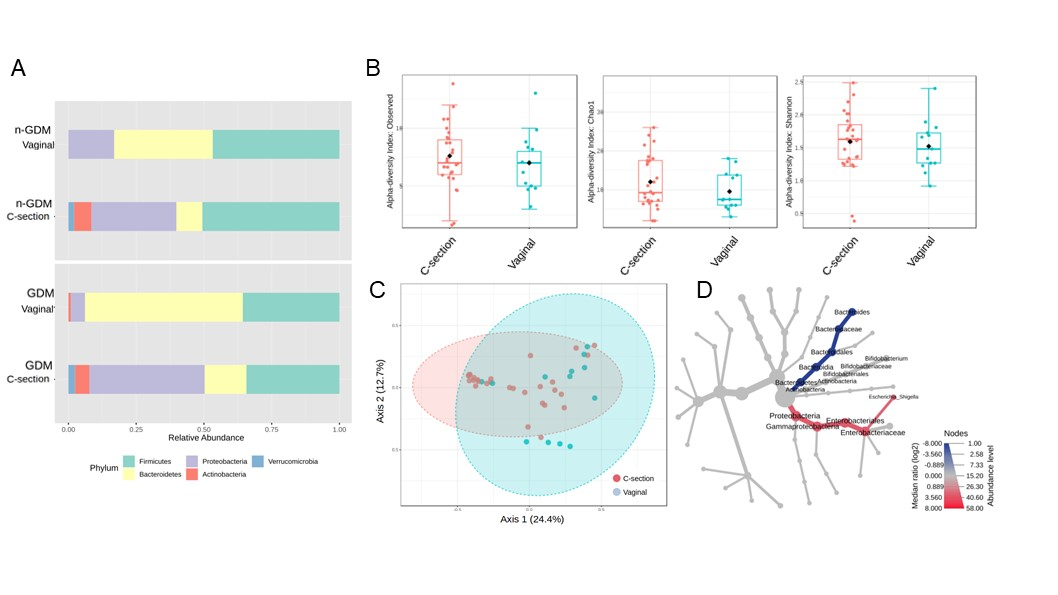

Supplement: S1 Fig — (a) Phylum-level composition (% relative abundances) among the study groups. (b) Observed features, Chao and Shannon index in C-section and vaginally born infants, no significant differences were observed between the groups; (c) Bray-Curtis index in C-section and vaginally born infants, no significant differences were shown amongst the groups. (d) Heat tree for pair-wise comparison. Those taxa that showed statistically significant differences were members of Proteobacteria in C-section group (red) and members of Bacteroidetes in vaginally born infants group (blue). The colour of each taxon indicates the log-2 ratio of the proportions observed in each condition. (TIF) [file pone.0302726.s001.tif]
